# Gankyrin as Potential Biomarker for Colorectal Cancer With Occult Liver Metastases

**DOI:** 10.3389/fonc.2021.656852

**Published:** 2021-07-28

**Authors:** Chengxing Wang, Xiaoping Li, Liangliang Ren, Changyi Ma, Meimei Wu, Weijun Liang, Jinglin Zhao, Shangren Li, Qunying Tan, Yuehua Liao, Lixia Sun, Xin Zhang, Yaoming He

**Affiliations:** ^1^Department of Gastrointestinal Surgery, Jiangmen Central Hospital, Affiliated Jiangmen Hospital of Sun Yat‐sen University, Jiangmen, China; ^2^Clinical Experimental Center, Jiangmen Key Laboratory of Clinical Biobanks and Translational Research, Jiangmen Central Hospital, Affiliated Jiangmen Hospital of Sun Yat-sen University, Jiangmen, China; ^3^Department of Radiology, Jiangmen Central Hospital, Affiliated Jiangmen Hospital of Sun Yat‐sen University, Jiangmen, China; ^4^Department of Pathology, Jiangmen Central Hospital, Affiliated Jiangmen Hospital of Sun Yat‐sen University, Jiangmen, China

**Keywords:** Gankyrin, colorectal cancer, occult, liver metastasis, biomarker

## Abstract

The majority of occult liver metastases cannot be detected by computed tomography (CT), magnetic resonance imaging (MRI) or other traditionally morphological imaging approaches since the lesions are too small or they have not yet formed cancer nodules. Gankyrin is a small molecular protein composed of seven ankyrin domains. In this study, the expression of Gankyrin in colorectal cancer (CRC) patients with liver metastases was investigated to determine its prognosis value. Gankyrin expression in CRC patients was initially analyzed using data from The Cancer Genome Atlas (TCGA) database and bioinformatics tools. RT-qPCR, western blotting, immunohistochemistry (IHC) and transwell migration and invasion assays were then performed to verify the expression and function of Gankyrin in CRC cell line, CRC tissues and matched non-tumor tissues of clinical patients. General clinicopathological information including TNM stage as well as preoperative and postoperative imaging results were collected. The main outcome indicator was overall survival (OS), referring to the length of time from surgery to either death or the last visit. Statistical analyses included chi-squared tests, Cox analyses, progression free survival (PFS) rates and OS rates. Elevated Gankyrin expression was confirmed in CRC patients. The upregulated Gankyrin expression was positively correlated with the progression of disease and liver metastasis in CRC patients. OS analysis revealed that prognosis was worse in CRC patients with high Gankyrin expression compared to those with low expression. CRC patients with higher Gankyrin expression also had a higher risk of occult liver metastases and a lower PFS rate. Therefore, Gankyrin can be used as a potential biomarker for early diagnosis of CRC with occult liver metastasis.

## Introduction

Metastasis is a biological characteristics of malignant tumors, and poses a challenge in clinical practice (1). In recent years, considerable progress has been made regarding the diagnosis and treatment of tumors, but tumor metastases are still responsible for 90% of cancer-related deaths. For colorectal cancer (CRC) patients, liver metastasis is the leading cause of mortality. It has been reported that 70% of patients who died of CRC had metastatic liver disease (2). The early diagnosis of liver metastasis in CRC strongly influences the choice of treatment and prognosis. However, most occult liver metastases cannot be detected by computed tomography (CT), magnetic resonance imaging (MRI) or other traditional morphological imaging techniques since the lesions can be very small or may not have yet formed cancer nodules (3). Therefore, a simple technique for early detection of occult liver metastases in CRC patients is an important measure to investigate to improve overall survival (OS) rates and quality of life.

Gankyrin, encoded by the *PSMD10* gene, is a small molecular protein composed of seven ankyrin domains, is involved in the formation of the 26S proteasome, and is related to the cell cycle and apoptotic processes in tumor cells (4, 5). Moreover, several studies have shown that Gankyrin mRNA or protein expression was upregulated in many types of cancer to varying degrees (6–8). The biological function and clinical significance of Gankyrin (*PSMD10*) in occult liver metastasis of CRC have not yet been thoroughly explored.

In this study, the elevated expression of Gankyrin and its positive correlation with disease progression as well as liver metastases in CRC patients were investigated. Gankyrin overexpression was shown to be an independent risk factor for the poor prognosis of CRC patients. Furthermore, CRC patients who did not have detectable liver metastases by conventional imaging examinations before their operation were followed up. It was discovered that patients with higher Gankyrin expression had a higher risk of liver metastasis and lower progression free survival (PFS) rates. Collectively, these data suggest that Gankyrin has the potential to serve as a biomarker for early detection of occult liver metastasis in CRC.

## Materials and Methods

### Bioinformatics Analysis

The Cancer Genome Atlas (TCGA) database of Gene Expression Profiling Interactive Analysis (GEPIA) platform (gepia.cancer-pku.cn) was used to analyze the PSMD10 mRNA levels in human CRC samples. The keywords “colorectal cancer” and “human” were input into the Gene Expression Omnibus (GEO) (www.ncbi.nlm.nih.gov/geo) search, and GEO series (GSE) was selected for data set analysis. Meta-analysis was performed on one group of microarray data through the ONCOMINE databases (www.oncomine.org). The data type was set to mRNA and filtered by “colorectal cancer”, “normal tissues” and “PSMD10”. Then, five data sets were selected for meta-analysis to compare the expression of Gankyrin in CRC and in matched normal colorectal tissues.

### Subjects and Specimens

One hundred and fifty patients with a pathologically confirmed CRC diagnosis from 2014 to 2016 at Jiangmen Central Hospital (Affiliated Jiangmen Hospital of Sun Yat-sen University) were enrolled in this study. Colorectal tissues were collected by colonoscopy or surgery and stored at the Clinical Experimental Center of Jiangmen Central Hospital. Among them, 65 were male and 85 were female. Ages ranged from 22 to 81, with an average age of 63 ± 12 years. The end time point of follow-up was February 2021 or the time of patient death. The death of a patient that was not tumor-related was classified as a loss of visit. The main outcome indicator was OS, which refers to the follow-up time from surgery to either death or the last visit. PFS, which was the period of time from the start of treatment to either worsening of the disease or patient death, was also recorded. This study was approved and supervised by the ethics committee of Jiangmen Central Hospital (decision no. JXY201839). Patients and volunteers all provided signed informed consent.

### Reverse Transcription Quantitative Polymerase Chain Reaction (RT-qPCR)

Total RNA from CRC tissues and paired non-tumor tissues was extracted using Minibest universal RNA Extraction Kit (Takara company, Japan), and RNA concentrations were quantified using a Nanodrop (Thermo company, Japan). mRNA was reverse transcribed using PrimeScript™ II (TaKaRaBio, Dalian, China) and the synthesized cDNA was subjected to RT-qPCR using the Golden SYBR Green Mix (2×) kit (Toneker Biotech, Shanghai, China) according to manufacturer’s instructions. Glyceraldehyde 3-phosphate dehydrogenase (GAPDH) was used as the internal control. Primer sequences used were: PSMD10-forward, 5′-AACTGACCAGGACAGCAGAACT-3′; PSMD10-reverse, 5′-ACAGCATTCACTTGAGCACCTT-3′; GAPDH-forward, 5′-TGAACGGGAAGCTCACTGG-3′; and GAPDH-reverse, 5′-TCCACCACCCTGTTGCTGTA-3′. The melting curves and E = 2^−△△Ct^ algorithm was analyzed using LightCycler software (Roche Diagnostics). The cycling conditions were as follows: initial denaturation at 95°C for 10 min, followed by 40 cycles of denaturation at 95°C for 1 min and annealing/extension at 56°C for 1 min. All samples were run in triplicate and normalized to internal controls. The fold-changes or relative PSMD10 expression levels were calculated based on the 2^−△△Ct^ method.

### Western Blot

Western blotting was performed according to our previous publication (9, 10). The antibodies used included rabbit anti-Gankyrin monoclonal antibody (1:1000; Abcam, USA), rabbit anti-GAPDH monoclonal antibody (1:1000; Abcam, USA) and goat anti-rabbit IgG-horse radish peroxidase (HRP)-linked secondary antibody (1:1500; Thermo Fisher Scientific, Inc. USA). Briefly, after blocking with 5% non-fat milk dissolved in Tris-buffered saline containing Tween-20 (TBST) at 37°C for 1 hour, the membranes were incubated with the anti-Gankyrin or anti-GAPDH primary antibody at 4°C overnight. The following day, the membranes were incubated with the secondary antibody at room temperature for 1 hour. The specific bands were developed using an enhanced chemiluminescence reagent in a gel imaging system, and the bands were analyzed using Image J software. A total of three independent experiments were performed.

### Immunohistochemistry (IHC)

The experimental operation was based on our previous publication (11). Paraffin embedded sections of CRC tissues were deparaffinized and rehydrated. Antigen retrieval was performed by immersion in citrate buffer (pH 6.0) for 15 min at 95°C prior to incubation with 0.3% hydrogen peroxide for 15 min at room temperature to block endogenous peroxidase activity. After rinsing with phosphate-buffered saline (PBS) and blocking with 5% normal goat serum (Thermo Fisher Scientific, Inc. USA) for 30 minutes at room temperature, primary anti-Gankyrin antibody (1:50;, Abcam, USA) was added to sections and incubated overnight at 4°C. The peroxidase-anti-peroxidase detection method was used for all sections, which were subsequently counterstained, dehydrated and mounted with a coverslip at room temperature. Yellow particles in the cytoplasm and/or nucleus were used to estimate the proportion of positive colorectal cells, and the intensity of staining was scored as negative (–), weak positive (+), medium positive (++) or strong positive (+++). The H-score was then calculated from multiplying the intensity score (from 0-3) by the percentage of positive cells (range 0-300) (12). Two experienced pathologists calculated the H-score according to a double-blind procedure.

### Transwell Migration and Invasion Assays

SW480 cells were transfected with lenti-virus expressing Gankyrin (Lenti-Gankyrin) or empty lentiviral vector (Lenti-vector). SW480-vector cells and SW480-Gankyrin cells were added to the upper chamber of the polycarbonate Transwell filter (pre-coated with Matrigel) in the BioCoatTM Invasion Chambers (BD Biosciences, San Jose, CA) respectively. RPMI1600 medium with 10% FBS was added to the lower chamber. The cells were allowed to migrate for 12 h at 37°C. After removing cells on the upper side of the transwell, the invading cells on the underside were fixed and stained with Giemsa solution. The stained invasive cells were photographed under a microscope. Three independent experiments were performed and the data are presented as the mean ± standard deviation (SD).

### Statistical Analysis

Statistical analysis was performed with Prism 8 (GraphPad Software Inc., San Diego, CA, USA) and SPSS 22.0 software (SPSS Inc., Chicago, IL, USA). Numerical data were expressed as the mean ± SD. The relationship between Gankyrin expression and the clinicopathological features was analyzed using the χ^2^ test. Survival analysis were performed using the Kaplan-Meier method and compared *via* the log-rank test. Variables were analyzed using univariate and multivariate Cox regression. A p value < 0.05 was considered statistically significant.

## Results

### Gankyrin Was Overexpressed in CRC

**Figure 1:**
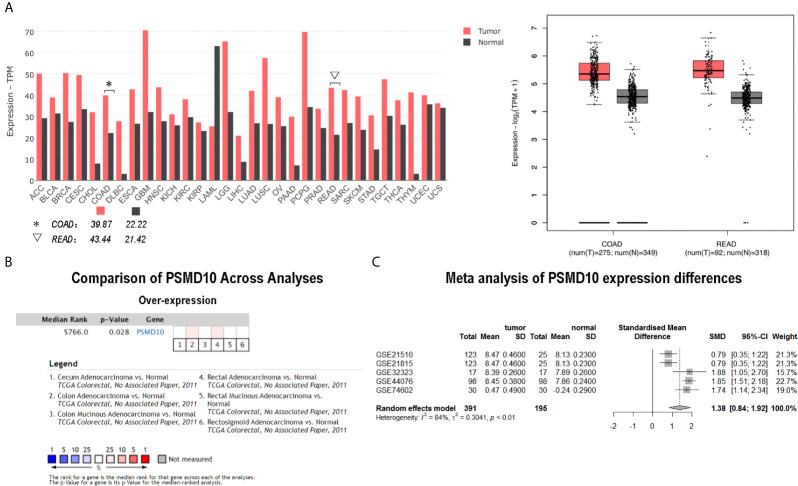
Bioinformatic analyses suggested that Gankyrin was upregulated in colorectal cancer (CRC). **(A)** The expression of Gankyrin (PSMD10) in human CRC tissues and matched non-tumor colorectal tissues was analyzed using The Cancer Genome Atlas (TCGA) database (COAD [colorectal adenocarcinoma] and READ [rectum adenocarcinoma])-based Gene Expression Profiling Interactive Analysis (GEPIA) platform. Box plot graph presented statistical results. **(B)** Using six independent studies from ONCOMINE database, comparison of the relative mRNA expression of Gankyrin in CRC with that in non-tumor colorectal tissue. **(C)** A meta-analysis of Gankyrin using five data sets is represented by forest plot.

**Figure 2:**
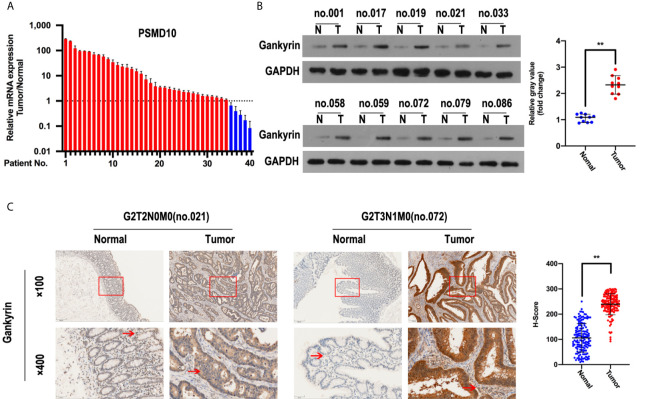
Gankyrin was overexpressed in colorectal cancer (CRC) tissues. **(A)** Gankyrin expression in CRC and matched non-tumor colorectal tissues were measured by RT-qPCR (n = 40), and values were presented as mean ± standard deviation (SD). **(B)** The expression of Gankyrin in CRC and matched non-tumor colorectal tissues was detected by western blot (n = 10), and numerical values were expressed as mean ± SD. Scatter plot displayed the difference of Gankyrin expression level. **(C)** Immunohistochemistry (IHC) staining of Gankyrin in CRC and matched non-tumor colorectal tissues (n = 150; scale bars, 50 μm and 200 μm), numerical values were expressed as mean ± SD. Scatter plot indicated the H-score of Gankyrin staining intensity. **p < 0.01.

The analysis using the TCGA, ONCOMINE and GEO databases showed that Gankyrin had higher mRNA expression in CRC tissues compared to matched non-tumor colorectal tissues (**Figures 1A–C**). To verify this finding, RT-qPCR and western blotting were used to measure the expression of Gankyrin. The RT-qPCR results revealed that the expression of Gankyrin mRNA in most CRC tissues was higher compared to non-tumor tissues (36/40; **Figure 2A**). Consistently, western blot analysis also showed that the expression of Gankyrin was increased in most CRC tissues (T) compared to matched non-tumor colorectal tissues (N; **Figure 2B**). In addition, the expression of Gankyrin was detected by IHC in all tissue specimens of CRC patients (n = 150). In most cases (146/150), the H-score of Gankyrin was higher in CRC tissues compared to matched non-tumor colorectal tissues (**Figure 2C**), which was in line with both the RT-qPCR and western blot data.

### The Overexpression of Gankyrin Was Positively Correlated With Liver Metastasis

**Figure 3:**
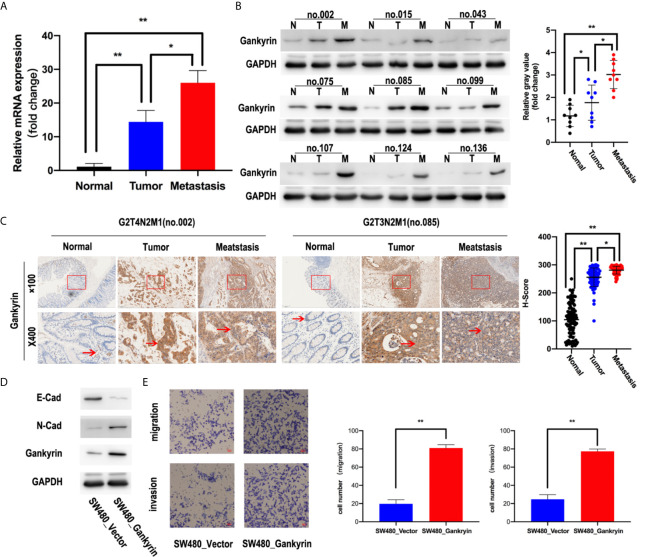
High Gankyrin expression was associated with liver metastasis of CRC. **(A)** Detection of Gankyrin expression in non-tumor colorectal tissues and matched CRC with or without liver metastasis tissues by RT-qPCR (n = 20). **(B)** The expression of Gankyrin in non-tumor colorectal tissues and matched CRC with or without liver metastasis tissues were detected by western blot (n = 9). Scatter plot showed the difference of Gankyrin expression level. **(C)** Immunohistochemistry (IHC) staining of Gankyrin in non-tumor colorectal tissues and matched CRC with or without liver metastasis tissues sections (n = 60; scale bars, 50 μm and 200 μm). Scatter plot indicated the H-score of Gankyrin IHC staining intensity. **(D)** SW480 cells were transfected with lenti-virus overexpressing Gankyrin (SW480-Gankyrin) or empty lentiviral vector (SW480-vector). 48 hours after transfection, total protein extracts were analyzed on Western blot for E-cadherin, N-cadherin and Gankyrin. **(E)** Matrigel invasion assay was used to measure CRC invasive ability, after 48 hours incubation, invaded cells were stained by hematoxylin and counted under a light microscope. The data represent the means of three independent experiments with SEM bars. In this series of statistical analysis, numerical value was expressed as the mean ± standard deviation (SD). *p < 0.05, **p < 0.01.

The expression of Gankyrin in paired non-tumor tissues and CRC tissues with or without liver metastasis were compared. Both the results of RT-qPCR and western blot revealed that the expression of Gankyrin was lower in non-tumor colorectal mucosa compared to paired CRC tissues (p < 0.05). In addition, the expression of Gankyrin in CRC tissues with liver metastasis was higher than in CRC tissues without liver metastasis (p < 0.05; **Figures 3A, B**). Similarly, a significant difference was verified by IHC (p < 0.05; **Figure 3C**). Furthermore, we found that epithelial markers of E-cadherin was drastically downregulated, but N-cadherin was dramatically upregulated in Gankyrin-transduced cells (SW480-Gankyrin) (**Figure 3D**). Moreover, the transwell matrix penetration assay showed that Gankyrin-overexpressing CRC cells enhanced the migration and invasion capacity (**Figure 3E**).These results collectively indicated that the elevated Gankyrin expression was positively correlated with liver metastasis of CRC, and it may promote CRC liver metastasis by inducing EMT.

### Elevated Expression of Gankyrin Suggested Poorer Outcomes for CRC Patients

**Figure 4:**
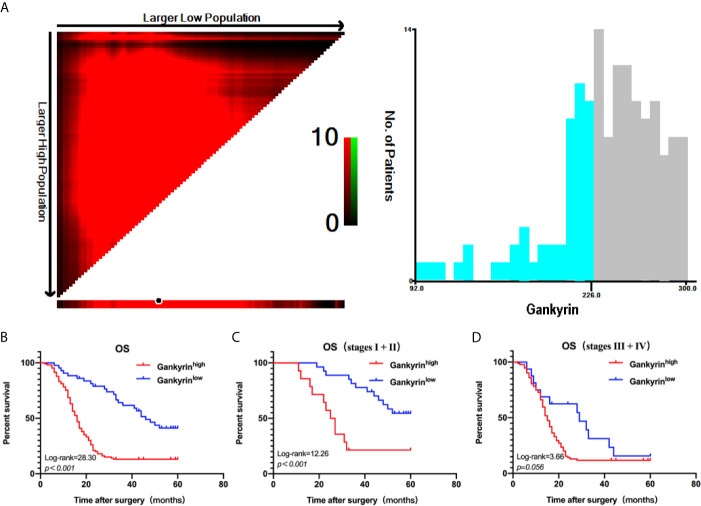
X-tile analysis of survival data. **(A)** The optimum cut-off points for Gankyrin was 226 (H-score) according to the X-tile program. **(B)** Kaplan-Meier overall survival (OS) analysis of colorectal cancer (CRC) patients with high and low expression levels of Gankyrin. **(C)** OS analysis of CRC patients with localized disease (stages I + II). **(D)** OS analysis of CRC patients with advanced disease (stages III + IV).

First, the relationship between high Gankyrin expression and the clinicopathological characteristics of 150 CRC patients were analyzed by univariate analysis. The results showed that the high Gankyrin expression was positively related to CEA level (p < 0.001), advanced TNM stage (p < 0.001), liver metastasis (p < 0.001) and lymph node metastasis (p < 0.001) in CRC patients (**Table 1**). Next, the risk factors for OS of 150 CRC patients were analyzed by univariate and multivariate Cox regression analyses. Multivariate Cox regression analysis showed that liver metastasis (p = 0.041) and a higher expression of Gankyrin (p < 0.001) were the risk factors associated with a poor prognosis in CRC patients (**Table 2**). Second, IHC was applied to examine the tissue expression of Gankyrin in the CRC patients (n=150), and Gankyrin expression was determined by the H-score. The optimal cut-off values for dividing patients into the low and high Gankyrin expression groups were determined by X-tile analysis, which indicated that the best cut-off value was 226 (H-score) using 5-year overall survival as the end point in the cohort (**Figure 4A**). In addition, Kaplan-Meier survival curves combined with the log rank test showed that the prognosis of CRC patients with high expression of Gankyrin was poor, and that the five-year survival rate was significantly reduced (p < 0.001; **Figure 4B**). In the localized group (stages I + II), the five-year survival rate of patients with high expression of Gankyrin was also lower compared to patients with low Gankyrin expression (p < 0.001; **Figure 4B**), but a comparison of the five-year survival rates in the advanced group (stages III + IV), the expression level of Gankyrin did not reflect a difference in survival (p = 0.056; **Figure 4C**).

**Table 1:** The relationship between Gankyrin expression and the clinicopathological features of 150 colorectal cancer (CRC) patients.

Characteristic	Sum	Number of Patients (%)		χ^2^ value	p value
		Gankyrin (High)	Gankyrin (Low)		
Age				0.018	0.895
≥ 55 years	65	46 (70.8)	19 (29.2)		
< 55 years	85	61 (71.8)	24 (28.2)		
Gender				0.350	0.555
Male	65	48 (73.8)	17 (26.2)		
Female	85	59 (69.4)	26 (30.6)		
Smoke				0.006	.0941
Yes	67	48 (71.6)	19 (28.4)		
No	83	59 (71.1)	24 (28.9)		
Drink				0.510	0.822
Yes	81	57 (70.4)	24 (29.6)		
No	69	50 (72.5)	19 (27.5)		
CEA				155.660	< 0.001
≥ 5 ng/mL	103	96 (93.2)	7 (6.8)		
< 5 ng/mL	47	11 (23.4)	36 (76.6)		
Tumor size				0.032	0.858
≥ 5 cm	75	53 (70.7)	22 (29.3)		
< 5 cm	75	54 (72.0)	21 (28.0)		
Tumor location				2.169	0.143
Colon	80	53 (66.3)	27 (33.7)		
Rectum	70	54 (77.1)	16 (22.9)		
TNM stage				62.071	< 0.001
I + II	41	14 (34.1)	27 (65.9)		
III + IV	109	93 (85.3)	16 (14.7)		
Family history				0.001	0.992
Yes	21	15 (71.4)	6 (28.6)		
No	129	92 (72.1)	37 (27.9)		
Liver metastasis				186.071	< 0.001
Positive	90	89 (98.9)	1 (1.1)		
Negative	60	18 (30.0)	42 (70.0)		
Lymph node metastasis				106.938	< 0.001
Positive	112	99 (88.4)	13 (11.6)		
Negative	38	8 (21.1)	30 (78.9)		
Total	150	107	43		

CEA, carcinoembryonic antigen. TNM, tumor node metastasis.

**Table 2:** Cox regression analysis of five years overall survival in 150 colorectal cancer (CRC) patients.

Predictor variables	Hazard Ratio	95% CI	p value
Age (≥ 55 years *vs* < 55 years)	0.880	0.628-1.232	0.456
Gender (Male *vs* Female)	0.829	0.592-1.161	0.275
Smoke (Yes *vs* No)	0.737	0.528-1.030	0.074
Drink (Yes *vs* No)	1.189	0.850-1.662	0.312
CEA (≥ 5 ng/mL *vs* < 5 ng/mL)	0.146	0.091-0.234	< 0.001
Tumor size (≥ 5 cm *vs* < 5 cm)	1.126	0.807-1.573	0.484
Tumor location (Colon *vs* Rectum)	1.243	0.889-1.737	0.203
TNM stage (III + IV *vs* I + II)	0.219	0.145-0.330	< 0.001
Family history (Yes *vs* No)	0.733	0.460-1.168	0.191
Lymph node metastasis (Positive *vs* Negative)	0.190	0.122-0.298	< 0.001
Liver metastasis (Positive *vs* Negative)	0.114	0.070-0.188	< 0.001
Gankyrin expression (High *vs* Low)	0.117	0.068-0.203	< 0.001
**Multivariable model**			
Liver metastasis (Positive *vs* Negative)	2.128	1.031-4.392	0.041
Gankyrin expression (High *vs* Low)	0.276	0.125-0.610	0.001

CI, confidence interval; CEA, carcinoembryonic antigen; TNM, tumor node metastasis.

### Overexpression of Gankyrin Suggested a High Risk of Liver Metastasis in CRC

**Figure 5:**
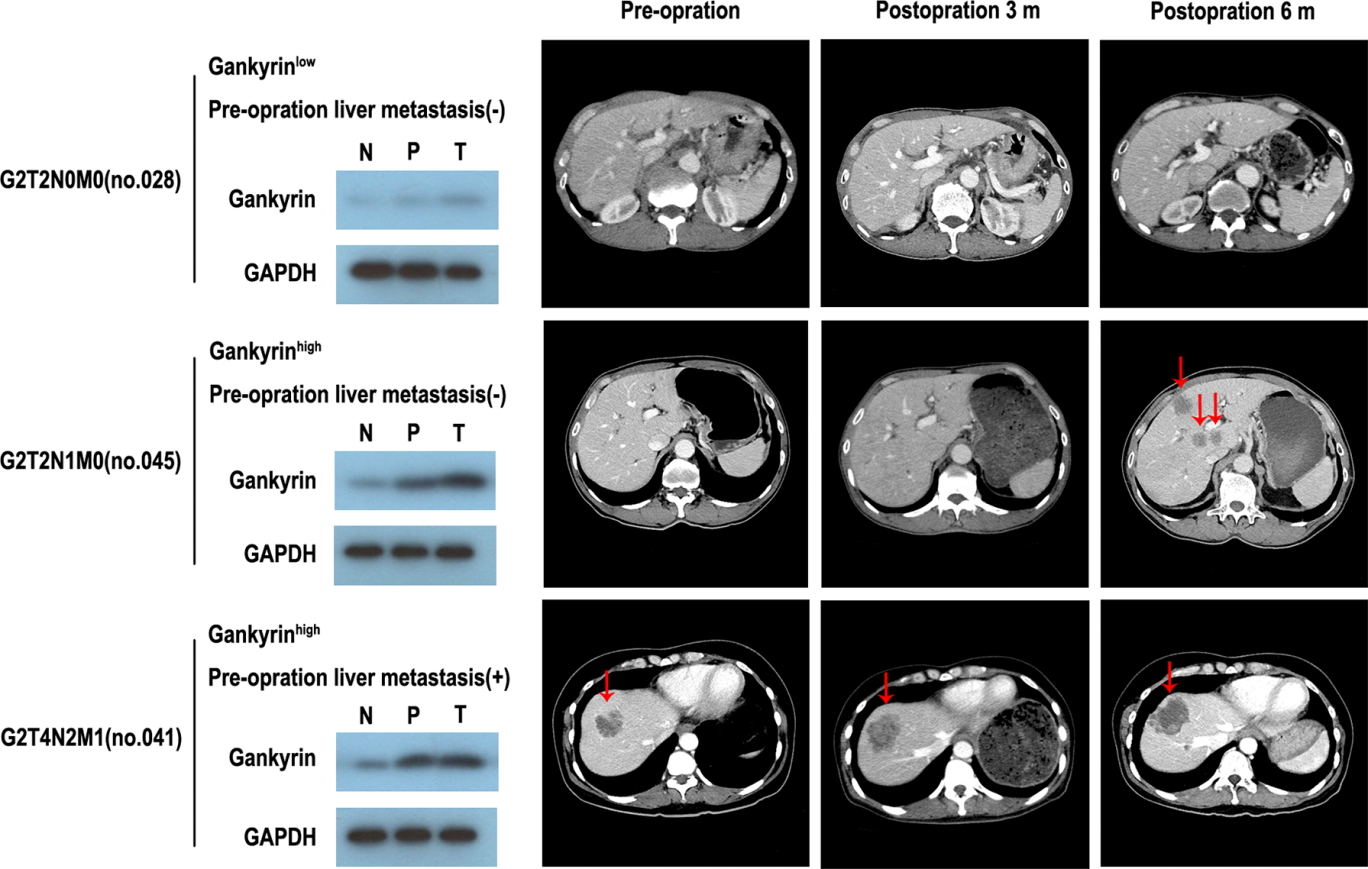
The expression of Gankyrin association with occult liver metastasis. Through the comparison of gankyin in non-tumor tissues, paracancerous tissues and cancerous tissues of colorectal cancer (CRC) patients, it is revealed that partial patients with high expression of Gankyrin are more likely to be in the state of occult liver metastasis, and then develop into CRC liver metastasis.

**Figure 6:**
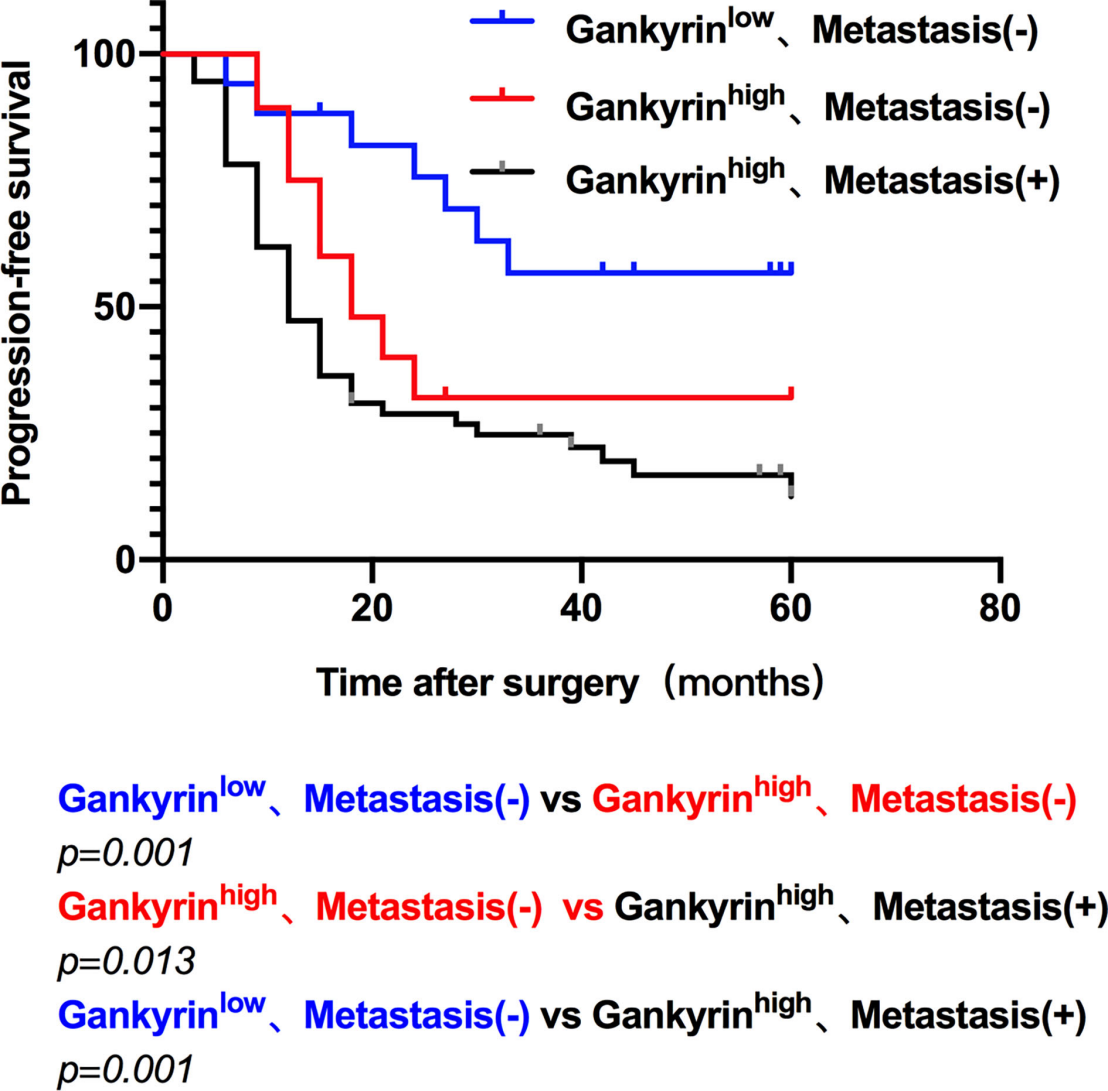
The progression free survival (PFS) of colorectal cancer (CRC) patients. The PFS was compared according to the expression of Gankyrin and the presence of liver metastasis, which characterized three groups: low Gankyrin expression without liver metastasis; high Gankyrin expression without liver metastasis; and high Gankyrin expression with liver metastasis.

The multivariate Cox regression analysis showed that high expression of Gankyrin was an independent risk factor for liver metastasis in CRC patients (p = 0.010; **Table 3**). During the follow-up of 60 CRC patients without liver metastasis before the operation, it was found that Gankyrin was strongly expressed in nine patients with CRC who did not show liver metastasis by preoperative CT or MRI (**Figure 5**). However, all nine of these patients developed detectable liver metastases within six months following the operation. The PFS of patients with strong Gankyrin expression was significantly shorter than that of patients with weak or no expression of Gankyrin (p < 0.001; **Figure 6**).

**Table 3:** Cox regression analysis of liver metastasis risk factors in colorectal cancer (CRC) patients.

Univariate Cox regression analysis	Overall survival		
	Hazard Ratio	95% CI	p value
Age (≥ 55 years *vs* < 55 years)	1.016	0.670-1.542	0.940
Gender (Male *vs* Female)	0.820	0.541-1.242	0.349
Smoke (Yes *vs* No)	0.674	0.447-1.023	0.064
Drink (Yes *vs* No)	1.123	0.740-1.704	0.585
CEA (≥ 5 ng/ml *vs* < 5 ng/ml)	0.013	0.002-0.074	< 0.001
Tumor size (≥ 5 cm *vs* < 5 cm)	0.948	0.627-1.433	0.800
Tumor location (Colon *vs* Rectum)	1.338	0.883-2.026	0.169
TNM stage (III + IV *vs* I + II)	0.015	0.003-0.089	< 0.001
Family history (Yes *vs* No)	0.670	0.384-1.168	0.158
Lymph node metastasis (Positive *vs* Negative)	0.018	0.003-0.110	< 0.001
Gankyrin expression (High *vs* Low)	0.012	0.002-0.090	< 0.001
Multivariate Cox regression analysis*	**Overall survival**		
	**Hazard Ratio**	**95% CI**	**p value**
Gankyrin expression (High *vs* Low)	13.573	1.854-99.384	0.010

CI, confidence interval; CEA, carcinoembryonic antigen; TNM, tumor node metastasis.

*Adjusted for variables from the above predictor variables using a backward stepwise cox proportional hazards model with a stay criterion of 0.10. p < 0.05 represents statistical significance.

## Discussion

At present, an early diagnosis of occult liver metastasis from CRC and an accurate judgement of prognosis remains clinically challenging (2, 13). To achieve individualized treatment for patients with different levels of risk, researchers are trying to find potential biomarkers that can accurately predict the outcome of CRC patients with liver metastasis. Gankyrin overexpression has been reported to be associated with the occurrence and progression of various cancers, including liver, pancreatic, kidney, endometrial, breast, and CRC (14–19).

Gankyrin can reduce the stability of the retinoblastoma (Rb) protein, causing dephosphorylation and inactivation of Rb, which activates E2 factor (E2F) transcription factors that induce the expression of genes related to cell cycle progression from G1 to S phase, and ultimately promotes cell division and growth that can lead to tumorigenesis (20). Gankyrin also interacts with cyclin-dependent kinase 4 (CDK4) to regulate the cell cycle and proliferation (21). In addition, Gankyrin can degrade tumor protein p53 (p53) through the ubiquitination pathway, resulting in decreased activity of this important tumor suppressor protein (22).

More importantly, many studies have shown that Gankyrin is expected to be a promising target for the treatment of a wide range of cancers involving metastasis in the future. Jin et al. found that Gankyrin was closely related to Ras-induced transformation (23). The study conducted by Man and colleagues revealed that Gankyrin is highly expressed in lung cancer cell lines and lung cancer tissues, and mediates phosphatase and tensin homolog (PTEN) degradation through an interaction with Rho GDP-dissociation inhibitor (RhoGDI). This in turn regulates the activation of phosphoinositide 3-kinase (PI3K)/Akt signaling to promote the occurrence of lung cancer (24). Bai et al. reported that Gankyrin is highly expressed in human CRC cell lines and tissues and can promote the migration of CRC cells by upregulating IL-8 signaling (25). Liu and colleagues have suggested that Gankyrin promotes tumor occurrence, metastasis and drug resistance by activating the β-catenin/c-myc signaling pathway in human hepatocellular carcinoma cell lines (26). Recently, He and colleagues found that Gankyrin promotes the invasion and progression of CRC by maintaining PI3K/GSK-3β/β-catenin signal activation (27). Taken together, these data indicate that the application of biomarkers for the early diagnosis of occult liver metastases from CRC is still in preliminary stages, and finding a suitable biomarker is important for current research.

In the present study, from analyses using the GEPIA platform of TCGA database, as well as the ONCOMIE and GEO databases, we found that Gankyrin mRNA expression was significantly upregulated in CRC tissues but not in matched non-tumor tissues. To verify these bioinformatics results, used RT-qPCR, western blotting, IHC and transwell migration and invasion assays were used to detect the expression and function of Gankyrin in CRC cell line, CRC tissues and matched non-tumor tissues. Additionally, Gankyrin was found to be highly expressed in CRC tissues experimentally and was even higher in CRC tissues from patients with known liver metastases. Therefore, we concluded that the expression of Gankyrin is positively correlated with liver metastasis, and it may promote CRC liver metastasis by inducing EMT. In addition, correlation analysis showed that high expression of Gankyrin and TNM staging were independent risk factors for CRC, and the high expression of Gankyrin may be related to liver metastasis of CRC. The OS analysis suggested that patients with high Gankyrin expression had shorter survival times and worse prognoses. Moreover, in CRC patients without detectable liver metastasis by preoperative CT and/or MRI, those patients with stronger Gankyrin expression were found to have a higher risk of developing detectable liver metastasis and had shorter PFS rates at follow up. At the first visit, some patients with CRC have a higher risk of liver metastasis or already have occult metastases that cannot be found by conventional imaging examinations such as CT and MRI. Since the routine preoperative evaluation cannot detect this state, there will be no additional examinations (that are typically more sensitive but also more costly) in the process of diagnosis and treatment, which leads to an incorrect evaluation of the disease staging in patients. We hope that we can take advantage of Gankyrin to evaluate patients with a higher risk of occult liver metastasis or identify patients who already have occult liver metastasis at their first visit, which can better guide the choice of treatment and the frequency of postoperative follow-ups. Taken together, our results suggest that Gankyrin is a promising biomarker to indicate occult liver metastasis in CRC patients when it is difficult to detect through imaging examinations.

Our study has some limitations. Firstly, this single center study only included a small cohort. We plan to further investigate the role of Gankyrin in the diagnosis of CRC with occult liver metastases through a multicenter study. Secondly, we need further research to explore the correlation between Gankyrin expression in CRC tumor tissue and peripheral blood to improve the clinical feasibility for its use as a biomarker. Finally, the Gankyrin signaling pathway in liver metastases stemming from CRC has not been fully elucidated. As such, further *in vivo* and *in vitro* experiments are required.

In conclusion, Gankyrin can be used as a biomarker to predict the progression and prognosis of CRC in patients, and has the potential to allow for early diagnosis of CRC occult liver metastasis. The measurement of Gankyrin expression, combined with carcinoembryonic antigen (CEA) detection and other clinical indicators, is expected to improve the individual evaluation and management of CRC patients at a high risk of occult liver metastasis. In addition, the precise mechanism whereby Gankyrin plays a regulatory role in liver metastasis of CRC requires further experimental study.

## Data Availability Statement 

The original contributions presented in the study are included in the article/supplementary material. Further inquiries can be directed to the corresponding author.

## Ethics Statement 

The studies involving human participants were reviewed and approved by Medical ethics committee of Jiangmen Central Hospital. The patients/participants provided their written informed consent to participate in this study. Written informed consent was obtained from the individual(s) for the publication of any potentially identifiable images or data included in this article.

## Author Contributions

CW and YH designed the study. CW, XL and YH wrote the manuscript. XL, XZ, CM, LR, MW, WL, SL, YL and LS contributed to collection of the data. YH, QT, XZ and JZ contributed to the critical revision of the manuscript. All authors contributed to the article and approved the submitted version.

## Funding

The present study was supported by grants from Guangdong Medical Research Fund (no. B2021057), Elite Young Scholars Program of Jiangmen Central Hospital (no. J202002) and Jiangmen Planned Project of Science and Technology (no. 2019020200050000723).

## Conflict of Interest

The authors declare that the research was conducted in the absence of any commercial or financial relationships that could be construed as a potential conflict of interest.

## Publisher’s Note

All claims expressed in this article are solely those of the authors and do not necessarily represent those of their affiliated organizations, or those of the publisher, the editors and the reviewers. Any product that may be evaluated in this article, or claim that may be made by its manufacturer, is not guaranteed or endorsed by the publisher.

## Abbreviations

CEA, Carcinoembryonic Antigen; CT, Computed Tomography; CRC, Colorectal Cancer; EMT, Epithelial-mesenchymal transition; GEPIA, Gene Expression Profiling Interactive Analysis; GEO, Gene Expression Omnibus; IHC, Immunohistochemistry; MRI, Magnetic Resonance Imaging; OS, Overall Survival; PFS, Progression Free Survival; Rb, Retinoblastoma; TCGA, The Cancer Genome Atlas.
